# Glaucoma: from pathogenic mechanisms to retinal glial cell response to damage

**DOI:** 10.3389/fncel.2024.1354569

**Published:** 2024-01-25

**Authors:** Jose A. Fernández-Albarral, Ana I. Ramírez, Rosa de Hoz, José A. Matamoros, Elena Salobrar-García, Lorena Elvira-Hurtado, Inés López-Cuenca, Lidia Sánchez-Puebla, Juan J. Salazar, José M. Ramírez

**Affiliations:** ^1^Ramon Castroviejo Ophthalmological Research Institute, Complutense University of Madrid (UCM), Grupo UCM 920105, IdISSC, Madrid, Spain; ^2^Department of Immunology, Ophthalmology and ENT, Faculty of Optics and Optometry, Complutense University of Madrid, Madrid, Spain; ^3^Department of Immunology, Ophthalmology and ENT, School of Medicine, Complutense University of Madrid, Madrid, Spain

**Keywords:** glaucoma, neuroinflammation, glial activation, microglia, astrocytes, Müller cells, ageing

## Abstract

Glaucoma is a neurodegenerative disease of the retina characterized by the irreversible loss of retinal ganglion cells (RGCs) leading to visual loss. Degeneration of RGCs and loss of their axons, as well as damage and remodeling of the lamina cribrosa are the main events in the pathogenesis of glaucoma. Different molecular pathways are involved in RGC death, which are triggered and exacerbated as a consequence of a number of risk factors such as elevated intraocular pressure (IOP), age, ocular biomechanics, or low ocular perfusion pressure. Increased IOP is one of the most important risk factors associated with this pathology and the only one for which treatment is currently available, nevertheless, on many cases the progression of the disease continues, despite IOP control. Thus, the IOP elevation is not the only trigger of glaucomatous damage, showing the evidence that other factors can induce RGCs death in this pathology, would be involved in the advance of glaucomatous neurodegeneration. The underlying mechanisms driving the neurodegenerative process in glaucoma include ischemia/hypoxia, mitochondrial dysfunction, oxidative stress and neuroinflammation. In glaucoma, like as other neurodegenerative disorders, the immune system is involved and immunoregulation is conducted mainly by glial cells, microglia, astrocytes, and Müller cells. The increase in IOP produces the activation of glial cells in the retinal tissue. Chronic activation of glial cells in glaucoma may provoke a proinflammatory state at the retinal level inducing blood retinal barrier disruption and RGCs death. The modulation of the immune response in glaucoma as well as the activation of glial cells constitute an interesting new approach in the treatment of glaucoma.

## Graphical Abstract

Glaucoma is a multifactorial disease characterized by the interaction of different molecular mechanisms that can induced the onset and contribute to the progression of the pathology, and in which the dysregulation of gene transcription and genetic predisposition can also play a key role. 
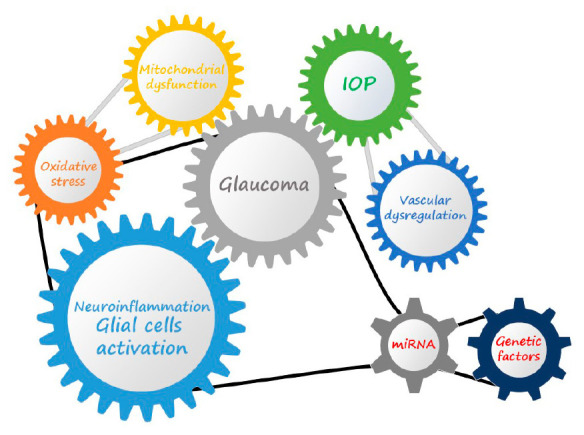

## 1 Glaucomatous optic neurodegeneration

### 1.1 General concepts

Glaucoma is a complex, chronic and multifactorial neurodegenerative pathology and is considered the leading cause of irreversible blindness (Weinreb et al., 2016; WHO, 2019).

In 2019, more than 60 million people were affected by glaucoma and are expected to reach nearly 95.4 million in 2030 (WHO, 2019). It is characterized by continued damage and progressive loss of RGCs and the retinal nerve fiber layer, as well as alterations of the optic disc and optic nerve head, eventually leading to progressive and irreversible constriction and loss of the visual field (Quigley and Broman, 2006; Weinreb et al., 2016).

Degeneration of RGCs and loss of their axons, as well as damage and remodeling of the lamina cribrosa constitute the main events in the pathogenesis of glaucoma (Giangiacomo and Coleman, 2009).

Different molecular pathways are involved in RGCs death, which are triggered and exacerbated as a consequence of a number of risk factors such as elevated IOP (Heijl et al., 2002; Nouri-Mahdavi et al., 2004), age (Leske et al., 2004; Lee et al., 2014), ocular biomechanics or low ocular perfusion pressure (Gordon et al., 2007; Quaranta et al., 2021).

Primary open-angle glaucoma is the most common form of glaucoma and is characterized by an alteration in the aqueous humor outflow pathways, which causes an increase in aqueous fluid outflow resistance leading to an increase in IOP. This ocular hypertension (OHT) leads to damage of the RGC axons in the optic nerve (ON) head and subsequently to concentric and progressive loss of RGCs (Mélik Parsadaniantz et al., 2020). Although most cases of glaucoma show elevated IOP values, there are patients with glaucomatous optic neuropathy whose pressure values are within the normal range (normotensive glaucoma), or who, despite elevated IOP values, do not present damage to the ON head (Jonas et al., 2014).

Current treatments are focused on OHT control, however, about 15–25% of these patients continue to exhibit disease progression despite correct IOP control (Tezel, 2013; Soto and Howell, 2014). Furthermore, in the case of normotensive glaucoma, elevated IOP cannot explain the neurodegenerative process (Wax et al., 2008).

The 70% of glaucoma cases are caused by elevated IOP, but it is clear that different molecular mechanisms are involved, such as oxidative stress, glutamate toxicity, neurotrophin deprivation, mitochondrial dysfunction, ischemia/hypoxia processes, protein misfolding, autoimmunity, dysfunction of autophagy processes, vascular dysregulation or neuroinflammation, which may also be responsible for RGC degeneration and death in cases of non-elevated IOP (Chang and Goldberg, 2012; Ramirez et al., 2017; Toft-Kehler et al., 2017; Duarte, 2021; Gutteridge, 2021).

### 1.2 Pathogenic mechanisms

#### 1.2.1 Intraocular pressure increase

Elevated IOP is one of the main risk factors in glaucoma and is the target of most current treatments. This increase in IOP is produced as a consequence of an increase in the outflow resistance of the aqueous humor as it is prevented from draining into the normal evacuation channels (Moreno et al., 2004).

Increased IOP leads to chronic stress on the ON head by mechanical compression of the ON head at the level of the lamina cribrosa, where the axons of the RGCs are more vulnerable to pressure-related changes. This is because the lamina cribrosa is structurally weaker than the sclera and more prone to distortion and posterior displacement (Quigley, 2005a,b; Burgoyne, 2011). In addition, at the level of the ON head, the axons of the RGCs make a 90° turn, which makes them particularly vulnerable to mechanical stress and alterations in axonal transport (Dias et al., 2022).

Analysis of the movements of the lamina cribrosa in glaucoma shows that the greatest mechanical damage is exerted on axons located in the peripheral part of the ON, which correlates with clinical findings showing that vision loss begins in the peripheral zone of the visual field (Burgoyne, 2011).

Although these physical changes in the lamina cribrosa may not be the cause of axonal loss in the early stages of glaucoma, stress and strain due to elevated IOP may accumulate, contributing to axonal damage and loss (Burgoyne et al., 2004; Burgoyne, 2011). The loss of elasticity of the lamina cribrosa with aging causes it to lose the ability to recover its normal configuration after normalization of IOP, increasing the prevalence of glaucoma with age (Albon et al., 2000).

The effect of this translaminar pressure gradient on axons leads to ischemia/hypoxia damage, blockade of axonal transport and deprivation of neurotrophic factors, ultimately resulting in apoptosis of RGCs (Nickells, 2007; Crish et al., 2010; Hirt et al., 2018). This pressure gradient alters retrograde and anterograde axonal transport, avoiding the cell bodies of RGCs to receive the contribution of neurotrophic factors such as brain derived neurotrophic factor (BDNF) or nerve growth factor (NGF) (Quigley, 1999; Almasieh et al., 2012; Križaj et al., 2014). As a consequence of this trophic deficit, abnormalities in the dendritic arborization of RGCs appear early after the increase in IOP (Weber et al., 2008).

In glaucoma, alterations in the ON head due to increased IOP also include reduced blood flow, oxidative stress, reactive gliosis, as well as remodeling of the extracellular matrix (Howell et al., 2007; Chidlow et al., 2011; Calkins et al., 2017; Tamm et al., 2017). Axonal remodeling, inflammation, glial activation and ultimately RGC death are partly dependent on intracellular Ca^2+^ (Crish and Calkins, 2011). Mechanical change-sensitive channels present in both the soma and dendrites of RGCs, such as P2X purinergic receptors, are activated by the tensional forces induced by increased IOP causing Ca^2+^ influx (Xia et al., 2012; Križaj et al., 2014). Intracellular Ca^2+^ triggers opening of Panexin-1 channels that allow massive ATP release. This ATP release by RGCs is associated with glial cell activation and increased expression of proinflammatory cytokines such as TNF-α, IL-1β, or IL-6 (Burnstock, 1999; Silverman et al., 2009).

Mitochondrial dysfunction can also occur as a consequence of both acute and chronic IOP elevation. Retinal hypoxia resulting from this increased pressure leads to increased production of reactive oxygen species (ROS), promoting intracellular lipid, protein, and DNA oxidation (Kamel et al., 2017).

#### 1.2.2 Mitochondrial dysfunction and oxidative stress

Energy in the form of ATP (Adenosine triphosphate) is required for neurotransmitter synthesis, synaptic transmission, restoring ionic gradients, Ca^2+^ buffering and bidirectional transport along axons (Ito and di Polo, 2017). Due to the absence of saltatory conduction in the axons of the unmyelinated part of the ON, a greater energy supply is required in this area, and there are a large number of mitochondria to alleviate this energy deficit (Bristow et al., 2002; Wang et al., 2003; Barron et al., 2004). This high energy consumption is necessary for the generation of action potentials and restoration of the resting membrane potential (Harris and Attwell, 2012). RGCs are able to maintain this level of consumption thanks to the abundant number of mitochondria present in their soma, axons and dendrites. The highest concentration is located at the level of the lamina cribrosa in order to protect this most vulnerable area of the retina and to ensure an adequate ATP supply (Minckler et al., 1976; Fan Gaskin et al., 2021).

Retinal ganglion cells have a low tolerance to mitochondrial damage. A decrease in the number of functional mitochondria or an accumulation of dysfunctional mitochondria may be the origin of an energy deficit in RGCs, also interfering with the maintenance of synaptic integrity (Li et al., 2004; Ito and di Polo, 2017; Toft-Kehler et al., 2017). Mitochondrial dysfunction leads to the release of multiple mitochondrial damage-associated molecular patterns (DAMPs). These molecules released by the dysfunctional mitochondria may result in an innate immune response contributing to the progression of the neuroinflammatory process in glaucoma (Duarte, 2021).

Mitochondrial dysfunction, in addition to being associated with alterations in mitochondrial DNA and deficient mitophagy, also occurs as a consequence of increased oxidative stress (Wilkins et al., 2014, 2017). Under normal conditions low levels of ROS production are counteracted by antioxidants such as glutathione synthetase, superoxide dismutase or catalase, but excess ROS can lead to lipid peroxidation and apoptosis by increasing mitochondrial membrane permeability and inhibition of the mitochondrial respiratory chain (Tezel and Yang, 2004; Li and Osborne, 2008; Inman et al., 2013; Iomdina et al., 2015).

Oxidative stress is a consequence of an imbalance between the production of ROS and antioxidant agents. Although ROS participate in signaling pathways of redox processes, they can also be responsible for cell damage, necrosis, or apoptosis, as a consequence of the oxidation process of macromolecules such as lipids, proteins, nuclear DNA, and mitochondrial DNA (Jarrett et al., 2008; Fan Gaskin et al., 2021). Dysregulation of ROS production and impaired ATP production by mitochondria lead to increased oxidative stress in RGCs, being an important immunostimulatory and cell death mechanism in glaucoma (Tezel, 2006; West and Shadel, 2017; Tang et al., 2019).

The accumulation of neurotoxic levels of glutamate is another effect of ROS excess. Glutamine synthetase (GS), responsible for transforming retinal glutamate to its non-toxic form, and glutamate transporter proteins are also altered by increased ROS in models of ocular hypertension (Moreno et al., 2004; Tezel et al., 2005). In addition, glutamate excess levels can also induce oxidative stress, being implicated in numerous neurodegenerative conditions (Cassano et al., 2016). Both glutamate accumulation and decreased glutamate transporters lead to apoptosis of RGCs (Hurley et al., 2022). Increased IOP causes elevation of glutamate levels increasing glutamate-induced oxidative stress in RGCs (Nucci et al., 2005). Glutamate analogs such as *N*-methyl-D-aspartate (NMDA) have been used in murine models to induce an increase in calcium and ROS levels and ultimately the death of RGCs (Seitz and Tamm, 2013).

Both altered intrinsic mitochondrial activity and exposure of RGCs and retinal neuroglia to extrinsic factors such as altered blood flow, hypoxia, nutrient deficits, or calcium dysregulation are associated with ROS production in glaucoma (Satilmis et al., 2003; Malone and Hernandez, 2007; McElnea et al., 2011; Yanagi et al., 2011; Vohra et al., 2019). In addition, mechanical damage to the ON head as a consequence of elevated IOP is also responsible for neuronal oxidative stress and lipid peroxidation, factors that increase the risk of neurodegeneration (Ko et al., 2005; Ferreira et al., 2010). In the anterior segment, exposure of the trabecular meshwork to high levels of ROS leads to increased normal aqueous humor outflow resistance, contributing to increased exposure of the retina and optic nerve head to elevated IOP values (Saccà et al., 2005; Duarte, 2021).

In the retina, Müller cells perform essential functions for the maintenance of homeostasis, many of which are related to their metabolism, so that energy restriction due to oxidative stress and mitochondrial dysfunction may contribute to the pathogenesis of glaucoma and the loss of RGCs (Kawasaki et al., 2000; Skytt et al., 2012; Toft-Kehler et al., 2017).

#### 1.2.3 Vascular dysregulation

Blood flow autoregulation in the retina involves vascular constriction or dilatation thereby increasing or decreasing resistance, thus maintaining a constant flow of nutrients in response to changes in perfusion pressure (Harris et al., 1998).

The optic nerve head is the site at which RGC axons are most vulnerable to potential ischemic events. Blood flow at this level is delicately regulated to ensure an adequate supply of oxygen and nutrients to RGC axons (Morgan, 2004). An insufficient blood supply to the optic nerve, either due to increased IOP or other vascular risk factors that may reduce ocular blood flow, will induce ischemia and hypoxia processes in the RGCs and their axons, contributing to the development of glaucomatous pathology (Flammer et al., 2002).

There are several indications postulating that reduced blood flow is one of the causes of glaucomatous pathology. Reduced ocular blood flow and its effects, such as hemorrhages at the level of the optic disc, considered hallmarks of glaucomatous processes especially in those poorly controlled, are often more pronounced in patients with normotensive glaucoma (Siegner and Netland, 1996; Healey et al., 1998; Drange et al., 2001). In addition to optic nerve hemorrhages, increased risk of venous thrombosis and retinal vasoconstriction are common in patients with glaucoma (Fan Gaskin et al., 2021).

Factors released by vascular endothelium or neural tissue contribute to the regulation of vascular tone in the retina. Nitric oxide (NO), a vasodilator factor, contributes to the autoregulation of retinal blood flow by protecting the nerve fiber layer against pathological stressors involved in glaucoma and ischemia (Toda and Nakanishi-Toda, 2007). On the other hand, the action of vasoconstrictors such as endothelin 1 (ET-1) or angiotensin II also participate in the changes in vascular tone. Alterations in NO activity and increased ET-1 expression, which could promote the weakening of the blood retinal barrier (BRB), have been observed in patients with glaucoma (Noske et al., 1997; Tezel et al., 1997; Tunny et al., 1998; Nicolela et al., 2003; Polak et al., 2007). Alterations in vascular autoregulation make the optic nerve head more vulnerable to decreased perfusion pressure, IOP increases and local metabolic demands (Moore et al., 2008).

**FIGURE 1:**
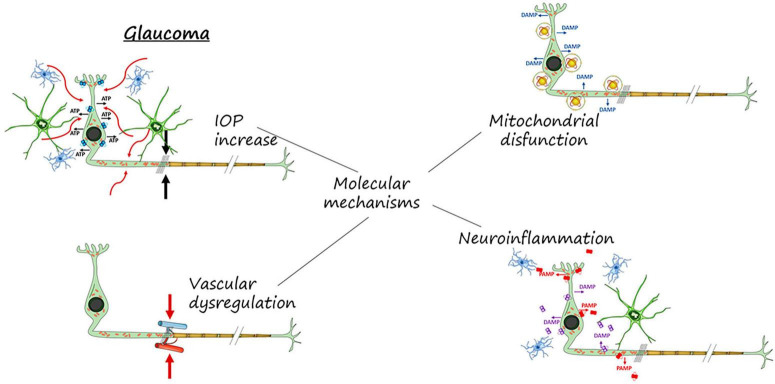
Molecular mechanisms involved in RGCs neurodegeneration and death. IOP increase, mitochondrial dysfunction, vascular dysregulation and neuroinflammation.

Systemically, there is an association between cardiovascular pathologies and the development of glaucoma. Both systemic hypertension and hypotension (Schmidl et al., 2011; Zhao et al., 2014; Levine et al., 2017; de Moraes et al., 2018; Skrzypecki et al., 2019), diabetes (Gerber et al., 2015; Song et al., 2016), previous hemodynamic seizures or increased blood viscosity are considered specific risk factors for glaucoma (Fan Gaskin et al., 2021; Figure 1).

### 1.3 MicroRNAs in glaucoma

MicroRNAs (miRNAs) are small non-coding ribonucleic acid (RNA) molecules that play a key role in post-transcriptional gene regulation and are involved in a variety of biological processes, such as cell growth, differentiation, development, and apoptosis (O’Brien et al., 2018; Saliminejad et al., 2019). Dysregulated miRNAs can modulate key pathways and processes that are involved in glaucoma, such as apoptosis, autophagy, neurogenesis, aging, extracellular matrix remodeling, oxidative stress, inflammation, and angiogenesis (Cannell et al., 2008). miRNAs are involved in the maintenance of aqueous humor (AH) balance, trabecular meshwork (TM) changes, and RGC apoptosis, and studies have shown altered expression patterns of miRNAs in both glaucoma patients and animal models (Cannell et al., 2008; Greene et al., 2022).

Retinal ganglion cells apoptosis and neuroinflammation are molecular pathways regulated by differentially expressed miRNAs in glaucoma. Altered levels of different miRNAs in AH samples from glaucoma patients may regulate target genes linked to apoptosis and inflammation, such as Bcl-2, caspase-3, IL-6, IL-8, TNF, and nuclear factor κB (NFκB) (Li et al., 2010; Su et al., 2017; Wang et al., 2018; Hindle et al., 2019; Ou-Yang et al., 2020; Youngblood et al., 2020; Tabak et al., 2021).

Extracellular matrix remodeling and autophagy are also miRNA-regulated processes differentially expressed in glaucoma. These processes are involved in the degradation and renewal of the extracellular matrix and cellular components of the TM, affecting outflow resistance and IOP (Gonzalez et al., 2014; Reinehr et al., 2022). Profiles expression of several miRNAs such as miR-518d, miR-143, and miR-660 negatively modulate pathways involved in cell proliferation, cell survival, extracellular matrix (ECM) remodeling, and adherents junction function (Jayaram et al., 2017).

Mitochondrial dysfunction and oxidative stress, lead to the accumulation of reactive oxygen species and the impairment of mitochondrial function, causing cellular damage and death. Several miRNAs such as miR-7, miR-24, miR-27a, miR-29b, and miR-4295, target various genes and pathways exerting antioxidant effects in TM cells, contributing to the prevention of oxidative damage in glaucoma (Tabak et al., 2021).

The retina is an important source of miRNAs. It expresses several miRNAs that control crucial processes such as RGC survival, axonal transport, synaptic transmission and neuroinflammation (Zuzic et al., 2019). In glaucoma, both proapoptotic (miR-16, −497, −29b and let-7a) and antiapoptotic (miR-27a) miRNAs show altered levels. Apoptotic miRNAs induce neurodegeneration through Toll-like receptor signaling, or by modulating the apoptosis regulator Bcl-2. The up-regulation of miR-27a and down-regulation of pro-apoptotic miRNAs suggests a shift toward a protective anti-apoptotic phenotype of their expression profiles (Jayaram et al., 2015). The up-regulation of miRNA-96 is related to RGCs activity and activation of the caspase pathway. miRNA-96 affects survival and increases apoptosis of RGCs through interaction with caspase-2 (Kang et al., 2015). On the other hand, down-regulation of miRNA-100 reduces RGC apoptosis and promotes nerve growth through the phosphorylation pathway, controlling the intrinsic apoptotic pathway responsible for impaired mitochondrial function (Chrysostomou et al., 2013; Kong et al., 2014).

Expression profiles of miRNAs are also implicated in the microglial reactivity and RGCs survival in response to ocular hypertension. miR-93 agomir reduces cytokine expression, microglial activation, inflammation, and RGCs loss, by targeting STAT3 to exert its effect on RGC death (Wang et al., 2021).

### 1.4 Genetic factors in glaucoma

Glaucoma exhibits significant heritability, with early onset forms inherited as autosomal dominant or Mendelian recessive traits and adult-onset disease inherited as complex traits. Genes involved in early onset glaucoma have been identified by large-family linkage analysis, whereas genes involved in adult-onset disease have been identified by genome-wide association studies (GWAS), usually requiring a large number of glaucoma cases and controls (Wiggs, 2015; Wang et al., 2022).

Linkage studies of familial and congenital glaucoma have shown the existence of genes with a significant effect on the disease, such as MYOC (myocilin) and OPTN (optineurin) for familial primary open-angle glaucoma (POAG) and CYP1B1 for congenital glaucoma (Stoilov et al., 1997; Stone et al., 1997). Variations in the MYOC gene sequence lead to the most common form, characterized by elevated IOP, whereas rare mutations in OPTN and copy number variations in TBK-1 (TANK-binding protein 1) can cause familial NTG (normal tension glaucoma) (Stuart et al., 2023).

Myocilin is expressed in various eye tissues such as the iris, retina, trabecular meshwork, and cornea (Huang et al., 2000). MYOC mutations are mainly associated with juvenile-onset POAG (Fingert et al., 2002; Fingert, 2011). The phenotype of MYOC-associated mutations is generally severe, IOP is usually very high and surgical treatment is often required (Fingert et al., 1999). Accumulation of MYOC in the trabecular meshwork caused by mutations leading to overproduction or reduced degradation is associated with increased IOP and, ultimately, with the development of glaucoma (Doucette et al., 2015).

Optineurin is expressed in several ocular tissues including the cornea, retinal ganglion cells, and the trabecular meshwork (Li et al., 1998). Among all OPTN mutations, E50K is the one most clearly shown to cause glaucoma. The E50K mutation in POAG patients is associated with an earlier age of onset, more advanced optic nerve cupping and a more frequent need for surgical intervention (Aung et al., 2005).

TBK1 plays an essential role in the regulation of inflammatory responses to foreign agents (Fingert, 2011). A duplication of the TBK1 gene has been identified in approximately 1% of normal tension POAG cases (Ritch et al., 2014; Awadalla et al., 2015; Liu and Allingham, 2017). TBK1 and OPTN regulate autophagy of damaged mitochondria (Matsumoto et al., 2015; Richter et al., 2016). Duplication of the TBK-1 associated with NTG patients could be related to autophagy dysfunction and RGCs loss (Tucker et al., 2014; Fingert et al., 2017).

Recently GWAS have implicated ABCA1 [coding for ATP-binding cassette, subfamily A (ABC1) member 1] in POAG (Wiggs, 2015). ABCA1 is expressed in glaucoma-significant ocular tissues such as the iris, ciliary body, retina, optic nerve head, optic nerve, and trabecular meshwork (Chen et al., 2014; Gharahkhani et al., 2014). It has been suggested that ABCA1 may regulate neuroinflammation and neurodegeneration in the mouse brain, which may play a role in retinal pathogenesis and POAG (Chen et al., 2014; Gharahkhani et al., 2014). ABCA1 has been implicated in retinal inflammation and retinal ganglion cell apoptosis, and ABCA1 has also been shown to play a role in IOP regulation through aqueous humor dynamics (Li et al., 2018; Hu et al., 2020).

## 2 Neuroinflammation

### 2.1 General concepts

Neuroinflammation is the inflammatory response that occurs in neural tissue in response to neuronal damage. This inflammatory process is characterized by the activation of resident glial cells, the release of proinflammatory cytokines and chemokines, and the recruitment and infiltration of peripheral cells into the CNS as a consequence of the breakdown of the blood-brain barrier (Heneka et al., 2015; Kelly et al., 2020). Neuroinflammation is a defensive mechanism that will initially protect the CNS by eliminating or inhibiting different pathogens (Wyss-Coray and Mucke, 2002).

This inflammatory response may initially exert a neuroprotective effect by promoting the repair of damaged tissue and the elimination of cellular debris, while sustained inflammation may exert a negative effect by inhibiting the regeneration of neuronal tissue (Kempuraj et al., 2016; Russo and McGavern, 2016). This sustained inflammatory process can be stimulated by both endogenous factors (genetic factors, protein aggregates) and environmental factors (infection, trauma, drugs). The activation of glial cells in chronic inflammatory processes may contribute to the progression of neurodegenerative processes (Glass et al., 2010; Kempuraj et al., 2016; Stephenson et al., 2018), with most studies in this field concluding that both astrocytes and microglia are causally involved in the pathogenesis and progression of neurodegenerative disorders (Sarlus and Heneka, 2017; Hickman et al., 2018; Yamanaka and Komine, 2018). Glial cells are essential for the maintenance of homeostasis in the CNS, both in physiological and pathological conditions, and may therefore be directly involved in the development and progression of neurodegenerative pathologies (Parpura et al., 2012). Interaction between glial cells and neurons means that dysfunction of either can lead to the development of harmful processes that can disrupt neuron-glia communication or compromise neuronal viability (Patel et al., 2019; Bernaus et al., 2020). Glial cell activation-mediated neuroinflammation or gliosis is one of the first events in response to damage in both the brain and retina (Soto and Howell, 2014; Ramirez et al., 2017).

### 2.2 Glial cell activation: gliosis

In response to changes in the tissue environment, stress, infections, or neurodegenerative pathologies that may compromise neuronal survival or even with ageing, glial cells have the capacity to adopt different reactive phenotypes. Glial activation occurs in order to restore tissue homeostasis, repair and neuroprotection of damaged tissue. In the CNS, astrocytes and microglia are responsible for the innate immune response and their reaction is classically characterized by two opposite activation phenotypes acting as neuroprotective or neurotoxic agents (Kwon and Koh, 2020).

Microglia have been classically divided into two activation phenotypes, M1 (classical activation phenotype) and M2 (alternative activation phenotype) (Luo and Chen, 2012). The M1 phenotype is characterized by the expression of pro-inflammatory cytokines, expression of antigen presentation markers and phagocytic capacity (Mosser and Edwards, 2008), while M2, although also showing phagocytic capacity, is characterized by the expression of anti-inflammatory cytokines and extracellular matrix remodeling for tissue repair (Edwards et al., 2006). The alternative M2 activation phenotype has been subdivided into three categories M2a, M2b, and M2c based on different inductors and functions (Fan et al., 2022). These three stages are biochemically similar but show different mechanisms of activation; the STAT6 (signal transducer and activator of transcription-6) activation pathway and IL-4 induction are associated with the M2a phenotype, and the STAT3 pathway and IL-10 induction are associated with M2c (Matsukawa et al., 2003; Maier et al., 2012). On the other hand, the activation pathway of the M2b phenotype, induced by the binding of lipopolysaccharide (LPS) or IL-1β immune complexes to receptors (CD16, CD32, or CD64) on activated microglia, is not yet well defined (Moehle and West, 2015; Yang et al., 2017). Recently, the existence of an M3 activation phenotype acquired by proliferative microglia in response to colony-stimulating factor-1 (CSF-1) and IL-34 has also been suggested (Walker and Lue, 2015).

Similar to microglia, two activation phenotypes are also identified in astrocytes; A1 (neurotoxic) and A2 (neuroprotective), which are also related to the production of proinflammatory or immunomodulatory mediators according to their polarization state (Stephenson et al., 2018). On the other hand, A2 astrocytes, are considered neuroprotective and increase the expression of neurotrophic factors such as BDNF, glial cell line-derived neurotrophic factor (GDNF) and NGF, which promote synaptic growth and repair, as well as neuronal survival (Lam et al., 2019).

Astrocyte polarization toward an A1 or A2 phenotype is mainly controlled by the NFκB, toll-like receptors (TLRs), mitogen-activated protein kinase (MAPK) and the Janus kinase/STAT3 (JAK/STAT3) signaling pathway (Ding et al., 2021). The NFκB and MAPK pathways are associated with the neuroinflammatory response of astrocytes and regulate the production of inflammatory cytokines, chemokines, extracellular matrix proteins, cell adhesion molecules, ROS, and inducible nitric oxide synthase (iNOS), which are involved in leukocyte infiltration, neuronal death, and demyelination (Colombo and Farina, 2016; Perez-Nievas and Serrano-Pozo, 2018). Activation of TLR ligands on astrocytes also leads to the inflammatory response in the CNS (Azam et al., 2019). The JAK/STAT3 signaling pathway is associated with the activation of A2 astrocytes phenotype, regulating functions such as reduction of leukocyte infiltration, myelin sparing, expression of anti-inflammatory cytokines and promoting neuronal survival (Colombo and Farina, 2016; Liddelow and Barres, 2017).

However, both astrocytes and microglia are now considered to have a wide spectrum of activation phenotypes, and the loss of their neuroprotective functions toward potentially neurotoxic roles is directly related to the stage and severity of the neurodegenerative process (Bachiller et al., 2018).

Microglial cells are able to protect neural tissue against harmful stimuli such as pathogen-associated molecular patterns (PAMPs) and DAMPs, which are recognized by microglia-expressed receptors such as TLRs, oligomerization domain nuclear receptors or viral receptors (Glass et al., 2010; Stephenson et al., 2018). In response to these stimuli, microglia express proinflammatory cytokines such as TNF-α, IL-1β, IL-16 and chemokines such as monocyte chemoattractant protein-1 (MCP-1) or IL-18, which are responsible for cell recruitment and removal of pathogenic substances (Glass et al., 2010; Hickman et al., 2018).

Proinflammatory mediators released by neuroinflammatory M1 microglia, such as C1q, IL-1α, IL-1β, or TNF-α, have the ability to induce activation of the A1 astrocyte phenotype, which will be responsible for a secondary inflammatory response (Saijo et al., 2009; Liddelow and Barres, 2017). Astrocytes increase the expression of membrane receptors for IL-17 and tropomyosin receptor kinase B (TrkB) for neurotrophins during neuroinflammation. Binding of IL-17 to its receptor induces the recruitment of NFκB activator 1 (Act1) as well as the production of proinflammatory cytokines (Qian et al., 2007). In turn, transforming growth factor-β (TGF-β) expression by astrocytes inhibits NFκB signaling, helping to modulate and reduce the neuroinflammatory response (Cekanaviciute et al., 2014).

Molecular and morphological changes during astrogliosis assessed by glial fibrillary acidic protein (GFAP) expression are directly related to the severity of astroglial reactivity, being GFAP overexpression a characteristic sign of neuroinflammation in neurodegenerative processes in the CNS (Sofroniew, 2009).

As part of innate immunity mediated by glial cells and in response to PAMP and DAMP patterns, activation of pattern recognition receptors (PRRs), including TLRs, also induces the phagocytic capacity of glial cells (Underhill and Ozinsky, 2002). Microglial cells are the main cells responsible for phagocytosis in the CNS (Wolf et al., 2017), and during inflammatory processes they increase the expression of complement receptors such as CR1, CR3 and CR4 and TLRs, associated with an increase in their phagocytic capacity (Alexander et al., 2008). The phagocytic capacity of microglia may be impaired during ageing or in some pathological situations, making it necessary the action of auxiliary cells with phagocytic capacity (Abiega et al., 2016; Gabandé-Rodríguez et al., 2020).

In this context, astrocytes, although they are cells with lower phagocytic capacity and less efficient than microglia, are able to trigger a compensatory mechanism in response to phagocytic dysfunction in microglia (Konishi et al., 2020, 2022). Astrocytes have TLRs (TLR2, TLR3, TLR4, TLR5, and TLR9), increasing their expression in the presence of pro-inflammatory stimuli, acquiring the ability to engulf cellular debris and damaged or dead neurons (Bsibsi et al., 2002; Bowman et al., 2003; Morizawa et al., 2017; Ponath et al., 2017).

Like astrocytes, Müller cells express TLRs (TLR2, TLR3, TLR4, TLR5, TLR7, and TLR9) under normal conditions, and their expression increases under pathological conditions (Kumar and Shamsuddin, 2012). Expression of TLRs gives Müller cells phagocytic capacity, and they are critical for the maintenance of homeostasis, removal of neuronal debris and retinal reorganization and regeneration (Bejarano-Escobar et al., 2017; Sakami et al., 2019).

Although neuroinflammation is initially a neuroprotective mechanism, its sustained effect over time can induce a neurotoxic effect, which is directly related to neurodegenerative processes (Hickman et al., 2018).

#### 2.2.1 Morphological changes

Morphologically, microglia have the ability to adopt different morphotypes depending on their state of activation, from quiescent (branched microglia) to hyperbranched, activated, amoeboid or rod microglia (de Hoz et al., 2013; Holloway et al., 2019; Li et al., 2019).

Microglial cells are distributed in a regular mosaic and are characterized by small, rounded somas, from which long, thin, highly branched processes emerge, which are continuously extending and contracting, facilitating their immunosurveillance function (Nimmerjahn et al., 2005). Branched microglia are able to detect and respond to biochemical and bioelectrical changes in the microenvironment, acquiring the hyperbranched phenotype, characterized by a greater complexity of the processes, being more abundant, longer, and thicker, emerging from thicker and more irregularly shaped somas (Karperien et al., 2013; Torres-Platas et al., 2014).

After exposure to harmful agents, hyperbranched microglia can adopt an activated phenotype which presents similar somas to hyperbranched microglia, but showing much fewer, thicker, and retracted processes (Torres-Platas et al., 2014; Sasaki, 2017). In this state of activation, microglia are able to proliferate, induced by the presence of factors released by reactive astrocytes such as M-CSF (macrophage colony-stimulating factor) and GM-CSF (granulocyte-macrophage colony-stimulating factor), or by different cytokines; IL-1, IL-3, IL-4, IL-5, IL-10, and TNF-α (Schilling et al., 2001), as well as migration to the site of damage, in order to interact with damaged neurons and remove cellular debris or dead cells (Karlstetter et al., 2010).

After sustained exposure to these agents, activated microglia change to an amoeboid state, with a more regular morphology characterized by thicker, rounded somas and with few or even absent processes (Karperien et al., 2013; Torres-Platas et al., 2014). Amoeboid microglia, with phagocytic capacity, migrate to the site of damage to phagocytose cellular debris or dead neurons (Davis et al., 2017).

Another morphological type of activation is the rod microglia characterized by an elongated soma with very few processes, with a length similar to that of branched microglia, located close to neurons, aligned with nerve fibers (de Hoz et al., 2013; Giordano et al., 2021). This microglial morphotype is associated with neurodegeneration and neuronal circuit rearrangement (Neumann et al., 2009; Perry and O’Connor, 2010). Rod microglia run parallel to damaged neurons and disrupted axons in order to limit neuronal damage (Ziebell et al., 2012). Once recruited to the site of injury, rod microglia have the ability to degrade the extracellular matrix, to promote the retraction of dystrophic axons and to disrupt synapses, in a phenomenon known as “synaptic stripping,” decreasing synaptic activity and metabolic requirement of the damaged neurons (Perry and O’Connor, 2010; Cao et al., 2012; Bachstetter et al., 2017).

At final stages before cell death by apoptosis, microglia adopt a dystrophic state with partially or completely fragmented cytoplasm with retraction, alteration, or even complete lack of cell processes (Streit et al., 2004). In this dystrophic stage, microglia have lower neuroprotective capacity and release higher amounts of pro-inflammatory cytokines. This microglia produces chronic inflammatory mediators associated with neuroinflammation and parainflammation. Dystrophic microglial proliferation is associated with several neurodegenerative diseases such as Alzheimer’s or Huntington’s disease (Simmons et al., 2007; Streit et al., 2009; Hoare and Narita, 2013; Shahidehpour et al., 2021).

Similar to CNS, different morphotypes of microglia in the retina appear as a consequence of exposure to different cytokines, chemokines, or DAMPs (Karlstetter et al., 2015).

Quiescent astrocytes during astrogliosis, similar to microglia, are able to acquire a reactive phenotype in response to neuronal damage, undergoing different morphological changes (Sofroniew, 2009). The main changes associated with astrogliosis are cellular hypertrophy, characterized by an increase in the thickness of the soma and its cell processes, retraction, and reorientation of its primary processes, together with an increase in the number of primary and secondary processes (Peng et al., 2014). Cellular hypertrophy is accompanied by increased expression of intermediate filament constituent proteins, including vimentin, nestin and mainly GFAP, which is the main component of intermediate filaments in adult astrocytes (Pekny and Pekna, 2004).

Astrocyte proliferation occurs after sustained exposure to damage, whether in the presence of a chronic neurodegenerative process, trauma, ischemic and infectious processes (Sofroniew, 2014). Proliferation is associated with the expression of cytokines such as IFN-γ, TNF-α, M-CSF, IL-1, IL-2, and IL-6 or chemokines such as CXCL10 and CCL5 (Bajetto et al., 2002; Mohri et al., 2006; Zhang D. et al., 2009).

Astrocyte proliferation and glial scar formation play an essential role in protecting and regenerating tissue by isolating it from the site of damage, where cytotoxic molecules are released (Liu et al., 2014). However, at later stages of tissue remodeling, they can also prevent normal axonal repair and regeneration processes (Menet et al., 2003; Vay et al., 2021). Factors released by astrocytes such as TGF-β and NGF are involved in the formation of these glial scars (Escartin and Bonvento, 2008).

Müller cells in the retina, similar to astrocytes, have the capacity to react in the presence of pathological conditions, injury, or inflammation, acquiring a reactive phenotype (Wang and Wong, 2014). The main characteristics of Müller cell gliosis are cell hypertrophy, with thickening of both somas and processes, proliferation, as well as increased expression of cytoskeleton constituent proteins such as vimentin or GFAP, and decreased expression of GS (Bringmann et al., 2009).

During gliosis, Müller cells will also contribute to the formation of glial scars, which can inhibit the remodeling and normal regeneration of damaged retinal tissue (Bringmann et al., 2009; Bringmann and Wiedemann, 2012). Increased GFAP expression, as well as the expression of extracellular matrix molecules and cell adhesion molecules, can inhibit normal neurite and axon growth, and thus neuronal regeneration (Lu et al., 2011; Bringmann and Wiedemann, 2012). Growth factors released by glial cells during reactive gliosis, such as basic fibroblast growth factor (bFGF), NGF, epidermal growth factor (EGF) and platelet-derived growth factor (PDGF), as well as glutamate or cytokines such as IL-1 or IL-2, are associated with Müller cell proliferation (Lewis et al., 1999; Kruchkova et al., 2001; Bringmann et al., 2006).

#### 2.2.2 Molecular changes

In the CNS, quiescent microglia, in response to proinflammatory cytokines from pathogen debris and damaged cells, show an activated phenotype expressing proinflammatory factors such as IL-6, TNF-α, IL-1β, NO and proteases, which have negative effects on neurodegenerative processes (Liddelow and Barres, 2017). The classical M1 activation phenotype, induced by the presence of IFN-γ, released by T cells, LPS or DAMP, is characterized by a microglia with the capacity to produce inflammatory cytokines (IL-23, IL-18, IL-12, IL-1β, IL-6, TNF-α, CCL2, and CXCL10), ROS, NO and matrix metalloproteinases (MMP9 and MMP3), as well as acting as an antigen-presenting cell preventing the entry of pathogens (Cherry et al., 2014; Liu et al., 2019).

On the other hand, anti-inflammatory factors such as IL-4, IL-10, IL-13, and TGF-β lead to the neuroprotective M2a phenotype of microglia promoting the release of factors such as arginase-1, CD206 (macrophage mannose receptor), insulin-like growth factor 1 (IGF-1), Ym1 and FIZZ1, associated with neuroprotection and tissue repair (Glass et al., 2010; Sica and Mantovani, 2012; Tang and Le, 2016; Liddelow and Barres, 2017). M2b phenotype activation induced by immune complexes and TLRs agonists increases the expression of IL-1β, CD86, suppressor of cytokine signaling 3 (SOCS3), IL-6, IL-10, involved in phagocytosis and removal of damaged tissue debris (Franco and Fernández-Suárez, 2015; Lan et al., 2017). Once the immune response is weakened, the expression of TGF-β, IL-10 and glucocorticoids induces the appearance of the M2c phenotype promoting tissue regeneration (Mantovani et al., 2004).

The expression of different surface channels and receptors on microglia and their interaction with pro- or anti-inflammatory molecules will influence the density, spatial distribution, activation state and morphotype, as well as the pathogenesis of the neurodegenerative process (Ma et al., 2019). For example, IL-4 expression by microglia suppresses the release of proinflammatory cytokines such as IL-6, TNF-α, or NO, contributing to the transition of microglia toward the neuroprotective phenotype (Park et al., 2005; Zhao et al., 2006). In the retina, branched microglia show high expression of P2RY12, which is associated with immunosurveillance functions, while amoeboid microglia express high levels of CD68, a classic marker of phagocytosis (Walker et al., 2014; Dubbelaar et al., 2018; Waller et al., 2019).

Similar to microglia, pro-inflammatory reactive astrocytes increase the expression of genes related to the complement cascade, inducing the production of factors such as TNF-α, IL-1β, and NO, which can exert a neurotoxic effect (Liddelow and Barres, 2017). The production of inflammatory cytokines such as TNF-α, IL-1β, IL-6, IL-12, IL-15, IL-17, and IL-23 contribute to exacerbate the inflammatory response in the CNS (Jensen et al., 2013). Chemokines such as CCL2, CCL5, CXCL1, CXCL9, CXCL10, and CXCL12, produced by astrocytes, promote the recruitment of macrophages, monocytes, T and B lymphocytes and neutrophils (Jensen et al., 2013). In addition, neurotoxic astrocytes release NO, ROS, vascular endothelial growth factor (VEGF), and vascular cell adhesion molecules (VCAM-1), which contribute to maintaining the inflammatory microenvironment, BBB rupture and leukocyte extravasation into the CNS parenchyma (Gimenez et al., 2004; Swanson et al., 2004; Argaw et al., 2012).

Reactive astrocytes also increase the expression of neurotrophic factors and neuroprotective thrombospondins (Liddelow and Barres, 2017). Astrocytes with a neuroprotective role produce ciliary neurotrophic factor (CNTF) and BDNF increasing neuronal survival (Lee et al., 1998; Ikeda et al., 2001). Expression of glutamate transporters such as glutamate aspartate transporter (GLAST) and glutamate transporter-1 (GLT-1), retinoic acid and glutathione protect neurons against oxidative stress, ON excitotoxicity, increase anti-inflammatory character and protect the BBB (Rothstein et al., 1996; Chen et al., 2001; Mizee et al., 2014). In addition, astrocytes produce molecules with anti-inflammatory effects on microglia and monocytes (Min et al., 2006; Kostianovsky et al., 2008). The presence of anti-inflammatory cytokines such as IL-10, IL-4, or IL-13 induces the appearance of neuroprotective reactive astrocytes, which in turn have the capacity to release IL-4, IL-10, and TGF-β (Oksanen et al., 2019).

Müller cells can play both a neurotoxic and a neuroprotective role during gliosis. Müller cells are capable of producing neuroprotective factors such as pigment epithelium-derived factor (PEDF) or VEGF, or cytokines such as IL-6, which contribute to restarting the cell cycle after damage, axonal regeneration, and neuronal survival (Yoshida et al., 2001; Harada et al., 2002; Unterlauft et al., 2012; Foxton et al., 2013; Galan et al., 2014). On the other hand, Müller cells are a source of proinflammatory cytokines such as IL-1β, TNF-α, and IL-6 (Coughlin et al., 2017). Complement protein C1q and NO expression in Müller cells is associated with inflammation and RGCs loss (Neufeld, 1999; Stasi et al., 2006). Activation of the NFκB signaling pathway and increased NGF expression induce TNF-α expression by Müller cells, contributing to neurotoxic effect (Lebrun-Julien et al., 2009). Expression of both NO and TNF-α exerts a neuronal death-inducing effect (Bringmann et al., 2006).

After retinal damage, increased expression of inflammatory factors such as MCP-1 by Müller glia promotes the recruitment of monocytes/macrophages and microglial cells to the site of damage, which release proinflammatory cytokines and oxygen free radicals, leading to neuronal apoptosis and retinal degeneration (Nakazawa et al., 2006a,^2007^; Hollborn et al., 2008).

Glial cells are also involved in the adaptive immune response, mediated by T and B lymphocytes. APCs express MHC-II on their surface and are responsible for presenting antigens to activated T-lymphocytes, which are able to recognize antigens, although not directly. Dendritic cells, B lymphocytes and monocytes/macrophages are considered professional APCs that constitutively have high expression of MHC-II molecules on their surface. APCs degrade uptaken antigens, via endolysosomal pathway, into smaller fragments that are presented bound to MHC-II (Campana et al., 2015). In addition, they also have co-stimulatory molecules on their surface such as CD80, CD86, and CD40, which are necessary for T-cell activation (Blum et al., 2013). The presence of these co-stimulatory molecules in APCs can be induced by the expression of pro-inflammatory molecules such as IFN-γ (Rostami et al., 2020).

Glial cells, under physiological conditions, have very low or even no MHC-II levels on their surface; only microglial cells have low MHC-II expression, while it is absent in astrocytes and Müller cells (Aloisi et al., 2000a). During gliosis and in response to severe inflammatory processes, increased oxidative stress or in the presence of pro-inflammatory molecules such as IFN-γ or TNF-α, glial cells increase the expression of MHC-II, acting as non-professional APCs (Aloisi et al., 2000b; Bringmann et al., 2009).

In addition, glial cells also show increased expression of co-stimulatory molecules, which are essential for the triggering of the adaptive immune response (Nelson et al., 2002; Rostami et al., 2020). Increased MHC-II expression on reactive glial cells is directly related to inflammatory processes in the CNS (Schetters et al., 2018; Rostami et al., 2020).

### 2.3 Neuroinflammation and glaucoma

Neuroinflammation in glaucoma has become an important factor due to the role of both the immune system and glial cells in the early stages of the disease (Tezel, 2013; Rizzo et al., 2017). Different studies show that glaucoma has important similarities with other neurodegenerative pathologies associated with inflammation such as amyotrophic lateral sclerosis, Alzheimer’s disease, Parkinson’s disease, or frontotemporal dementia (Ramirez et al., 2017; Fernández-Albarral et al., 2019b). The A1 phenotype of astrocytes increases the expression of genes related to the complement cascade and neurotoxins, leading to a neurotoxic effect. In addition, A1 astrocytes induce loss of synaptogenesis and neuronal and oligodendrocyte death (Liddelow and Barres, 2017).

Different genes involved in inflammatory processes increase their expression in both the retina and the glaucomatous optic nerve head (Ahmed et al., 2004; Yang et al., 2007). The first pathways involved in increasing gene expression are TLR-mediated signaling pathways, for example, heat-shock proteins (HSPs) induce an increase in major histocompatibility complex class II (MHC-II) expression and cytokine production (Luo et al., 2010). Second, activation of the nuclear factor kappa B (NFκB)-mediated pathway causes an increase in the expression of cytokines belonging to the IL-1 family that in turn promote the release of a cascade of inflammatory cytokines such as TNF-α and IL-6 (Rolle et al., 2021). In the optic nerve of patients with glaucoma, elevated levels of both TNF-α and Fas ligand (FasL), a protein with proapoptotic capacity, are observed, and both are linked to the pathogenesis of glaucoma (Yan et al., 2000; Tezel et al., 2001; Sawada et al., 2010; Gregory et al., 2011).

The onset of the inflammatory process in glaucoma is triggered by impaired communication between RGCs and glial cells, inducing the release of proinflammatory mediators, such as ROS, NO, TNF-α, and IL-1β (Nakazawa et al., 2006b; Luo et al., 2010; Chua et al., 2012; Tezel et al., 2012; Madeira et al., 2015).

The marked increase in TNF-α expression as a result of proinflammatory imbalance in glaucoma is associated with RGCs loss (Yan et al., 2000; Tezel et al., 2001). This increase is also responsible for the alteration of mitochondrial function, reducing ATP production and increasing the production of ROS, contributing to the activation of the NFκB signaling pathway, and to the production of proinflammatory signals. This neuroinflammatory stimulation in turn promotes mitochondrial dysfunction of RGCs, constituting a cycle of neuronal damage (Wilkins et al., 2017).

Both macroglia (astrocytes and Müller cells) and microglia, as well as infiltrating monocytes, are involved in the neuroinflammatory process and in the immune-mediated neurodegenerative process of RGCs that occurs in glaucoma, producing proinflammatory cytokines and complement components (Howell et al., 2012; Nickells et al., 2012; Tezel, 2013; Soto and Howell, 2014). The vision loss process in glaucoma is also related to functional and distributional changes of retinal glial cells during reactive gliosis processes (Russo et al., 2016).

Glial cells have the ability to recognize DAMPs and PAMPs which are expressed on the surface of damaged cells or can be released into the extracellular space once the processes of apoptosis and necrosis have been triggered. Among the main DAMPs associated with RGC neurodegeneration are HSPs, uric acid and high-mobility group box (HMGB) proteins (Tezel and Wax, 2000; Rifkin et al., 2005; Glass et al., 2010; Yu et al., 2010; Zhu et al., 2011).

Astrocytes and Müller cells in response to glaucoma-associated stress signals undergo an important transformation process during reactive gliosis (Wang et al., 2002; Bringmann et al., 2006; Seitz et al., 2013; Lahne et al., 2020; Fernández-Albarral et al., 2022) showing both morphological changes and a wide spectrum of molecular and functional modifications (Johnson et al., 2007; Nikolskaya et al., 2009; Qu and Jakobs, 2013; Tehrani et al., 2016; Quillen et al., 2020; Fernández-Albarral et al., 2021).

**FIGURE 2:**
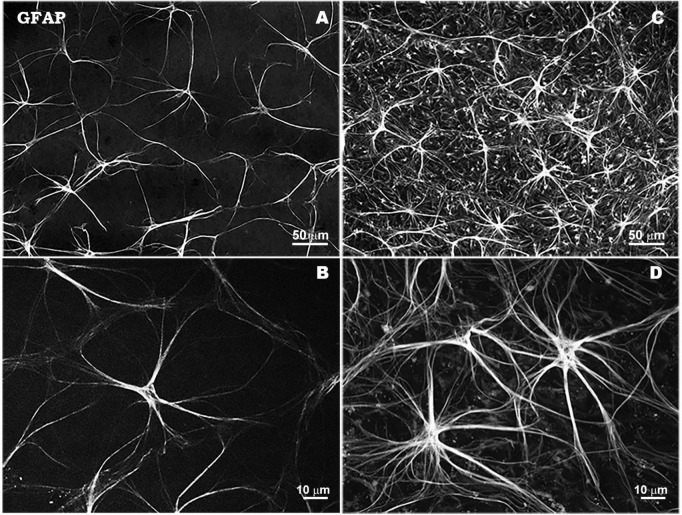
Morphological characteristics of astrocytes in response to elevated IOP in a laser-induced ocular hypertension experimental model in mouse. Astrocytes in hypertensive retina show an overexpression of GFAP immunostaining, a thickening of their soma size and an increase in the number and thickness of their processes. Retinal whole-mount. GFAP immunostaining. Naïve retina **(A,B)**. Hypertensive retina **(C,D)**. Images high magnification **(B,D)**. Modified from Fernández-Albarral et al. (2022).

Reactive astrocytes in glaucoma show an increase in GFAP expression, an increase in the size of their cytoskeleton and a larger extension of their cellular processes, as well as migrate to the site of damage (Tezel et al., 2001; Inman and Horner, 2007; Hernandez et al., 2008; Dai et al., 2012; Fernández-Albarral et al., 2022). Hypertrophic astrocytes maintain the integrity of neuronal tissue and form a barrier that isolates healthy neurons and protects them from neuroinflammatory signals, although at the same time they activate a survival mechanism that may limit the neurotrophic, bioenergetic and antioxidant supply to RGCs. This alteration of its functions together with astrocyte-mediated remodeling of the extracellular matrix and profibrotic processes amplify the biomechanical and vascular stress associated with glaucoma occurring in the optic nerve head (Roberts et al., 2010; Burgoyne, 2011; Dai et al., 2012; Figure 2).

Mechanical stress induced by increased IOP in glaucoma results in increased EGF receptor expression following astrocyte activation, as well as increased levels of TNF-α, matrix metalloproteinases (MMPs), endothelins, and nitric oxide synthase 2 (NOS-2), ultimately leading to a neurodegenerative response (Liddelow et al., 2017; Lin et al., 2019). In the course of this astrogliosis in glaucoma, there is also increased expression of the phagocytosis-related Mac-2 gene and ET-1 by astrocytes, demonstrating both their role in phagocytosis of damaged or dead RGC debris and their involvement in glaucoma-associated vascular dysregulation (Nickells et al., 2012; Chong and Martin, 2015).

Müller cells are one of the first glial cells to respond to glaucoma-associated stress signals such as increased IOP (Wang et al., 2002). In response to increased IOP, Müller cells increase the expression of both GFAP and GS (Zhang S. et al., 2009). Activation of Müller glia in glaucoma occurs with the objective to maintain neuronal tissue integrity and homeostasis by increasing the expression of receptors associated with neuronal growth and survival, increasing levels of extracellular glutamate uptake, thereby protecting RGCs from glutamate excitotoxicity, and releasing NGF (Newman and Reichenbach, 1996; Bringmann et al., 2009).

On the other hand, their chronic activation induces the release of TNF-α, IL-1, and NO that contribute to RGC degeneration and death. Moreover, this chronic activation of Müller cells impairs the survival of RGCs through activation of cell death receptors and alteration of retinal potassium and water homeostasis (Bringmann et al., 2005, 2006; Iandiev et al., 2008; Xu et al., 2009; Garcia et al., 2014). Furthermore, in this context Müller cells release ROS and prostaglandin E2 that also contribute to the apoptosis of RGCs (Gao et al., 2013; García-Bermúdez et al., 2021).

Mature microglial cells take part in inflammatory processes being activated by DAMPs released by neural tissue cells and also by astrocytes and even by microglia themselves (Rifkin et al., 2005; Yu et al., 2010; Zhu et al., 2011). Among the different DAMPs associated with inflammation, HSPs are released by RGCs in response to increased IOP and Tenascin C (TN-C) increases its expression in astrocytes and is responsible for TLR activation (Luo et al., 2010; Howell et al., 2012). Another mechanism induced by damaged neurons that leads to glial cell activation is the release of HMGB proteins, which will bind to CD11b receptors present on glial cells, stimulating the release of inflammatory cytokines and promoting the acquisition of reactive phenotypes (Nakazawa et al., 2006b).

**FIGURE 3:**
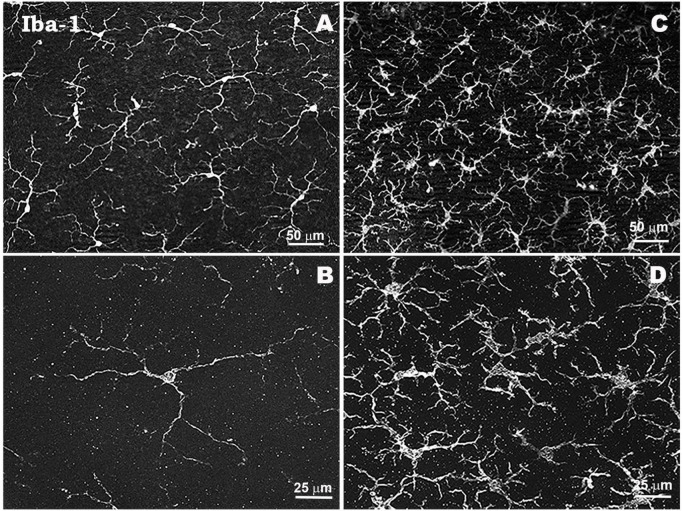
Morphological characteristics of microglial cells in response to elevated IOP in a laser-induced ocular hypertension experimental model in mouse. Microglia in hypertensive retina show proliferation phenomena, thickening of their soma and retraction and thickening of their processes. Retinal whole-mount. Iba-1 immunostaining. Naïve retina **(A,B)**. Hypertensive retina **(C,D)**. Images high magnification **(B,D)**. Modified from Ramírez et al. (2020a).

In glaucoma, reactive microglial cells initially phagocytize the debris of damaged or dead RGCs, contributing to the maintenance of a toxin-free microenvironment, and also release neurotrophic factors such as BDNF or CNTF, providing neuroprotection and promoting tissue neuroregeneration (Silverman and Wong, 2018; Alqawlaq et al., 2019). During the neuroinflammatory process in glaucoma, chronic activation of microglial cells leads to the release of cytokines and chemokines such as complement factors, TNF-α, IL-6, IL-1β, iNOS, NO, among others, which contribute to amplify the inflammatory response, induce morphological changes of different microglial phenotypes and contribute to apoptosis of RGCs (Ebneter et al., 2010; Bosco et al., 2012; Nickells et al., 2012; Wang et al., 2016; Fernández-Albarral et al., 2019a; Ramírez et al., 2020a; Fernandez-Albarral et al., 2022). In response to elevated IOP, the microglia exhibit different signs of activation, such as proliferation and migration to areas of damage, retraction, reorientation, and hyper-ramification of their processes, and the appearance of amoeboid- and rod-like microglia with macrophagic capacity, related to RGC degeneration (Ramírez et al., 2020a; Fernandez-Albarral et al., 2022; Figure 3).

Apoptosis is considered the main death pathway of RGCs in glaucoma and can be triggered either by signals from the cell soma, from their axons or from signals received from the extracellular environment (Quigley, 1999). Microglial and macroglial cells are involved in the activation and maintenance of apoptotic processes in glaucoma (Tezel, 2013). Increased IOP induces an inflammatory response of the glial population in the retina with the primary objective to isolate and resolve neural tissue damage by providing growth factors and metabolites to damaged RGCs, however, the chronification of this glial inflammatory response can affect glial cell function, neuronal support and extracellular glutamate buffering capacity (Martin et al., 2002), making RGCs and their axons more vulnerable and ultimately more prone to death by apoptosis (Neufeld and Liu, 2003; Weinreb and Tee Khaw, 2004; Quigley, 2005a,b; Tezel, 2011, 2013).

In addition to innate immunity, stress signals and tissue damage associated with glaucoma together with alterations resulting from mitochondrial dysfunction may also induce an adaptive immune response. The inflammatory microenvironment in glaucoma enriched by cytokines, chemokines and adhesion molecules released by glial cells is a supportive environment for glia-lymphocyte interactions and antigen presentation for an enlarged immune response (Tezel, 2022). Increased expression of MHC molecules and TLRs by glial cells allows them to participate in both innate and adaptive immunity (Yang et al., 2001; Luo et al., 2010). In glaucomatous patients, chronically activated microglial cells show increased MHC-II expression and GFAP-positive astrocytes show strong labeling with HLA-DR (human MHC-II molecule type) (Yang et al., 2001; Ramirez et al., 2017).

The increased expression of MHC-II by glial cells in both human and experimental animal models of glaucoma demonstrate the ability of these cells to act as antigen presenting cells (APCs) and stimulate an adaptive immune response (Yang et al., 2001; Ebneter et al., 2010; Howell et al., 2011; Gramlich et al., 2013; Chidlow et al., 2016). This along with the expression of proinflammatory cytokines leads to increased antigenic stimulation of T-lymphocytes. TNF-α expression by activated T cells is associated with other neurodegenerative CNS disorders and may be one of the causes of retinal changes in glaucomatous patients (Yang et al., 2011). In addition, alterations in vascular barriers, associated with glaucoma and ageing, facilitate the migration of T lymphocytes through the BRB and their contact with resident glia and autoantibodies generated by local antigens, promoting RGC damage through the toxicity of released proinflammatory cytokines (Wax et al., 2008; Gramlich et al., 2015; Chen et al., 2018).

The complement system, as part of innate immunity, plays an important role in immunosurveillance and is involved in the CNS inflammatory response to stress and damage (Tezel, 2013; Williams et al., 2016).

Associated with innate and adaptive immune responses, complement activation in glaucoma may contribute to neuroinflammation and RGCs degeneration. The complement protein C1q acts as a pattern recognition molecule tagging synapses and damaged neurons, thus initiating the classical complement activation pathway (Tezel, 2022). This complement activation appears to be important in the synaptic degeneration of RGCs in glaucoma, in a similar process to that occurring in the elimination of unwanted synapses during neuronal development (Stevens et al., 2007; Williams et al., 2016). Complement activation leads to cleavage of complement factor C3 and activation of the membrane attack complex (MAC). MAC is involved in the processes of cell lysis and necrotic death, and C3 is also associated with the processes of apoptosis (Fishelson et al., 2001; Rus et al., 2005; Harvey and Durant, 2014).

High concentrations of C5, an essential protein for MAC genesis, as well as MAC are associated with RGC apoptosis and axonal degeneration in the optic nerve head and pharmacological inhibition of the complement cascade reduces both apoptosis and MAC levels (Jha et al., 2011; Howell et al., 2013; Soto and Howell, 2014). High concentrations of C3, present in both RGCs and in the nerve fiber layer, are also associated with RGC and axon degeneration, and its inhibition under chronic elevated IOP leads to neuroprotection, suggesting a role in the development and progression of glaucoma (Kuehn et al., 2006; Bosco et al., 2018).

Increased expression of factors associated with complement activation such as C1q, C3, C5, and MAC are present in both glaucomatous patients and experimental animal models of glaucoma, noting that this increase occurs very early in response to IOP elevation (Stasi et al., 2006; Stevens et al., 2007; Howell et al., 2011; Johnson et al., 2011).

### 2.4 Neuroinflammation and ageing

Ageing is a natural and irreversible progressive phenomenon characterized by both anatomical and functional decline. Although it is a process that can occur in different ways in each individual, several common features of the ageing process have been defined, including genomic instability, telomere attrition, epigenetic alterations, loss of proteostasis, mitochondrial dysfunction, cellular ageing, stem cell depletion and altered intercellular communication, among others (López-Otín et al., 2013).

Among all, genomic instability and DNA damage constitute one of the main biological processes associated with ageing, leading to mitochondrial dysfunction, accumulation of ROS and cellular decline (Schumacher et al., 2021). In the CNS during ageing and due to its high energy requirement, a greater accumulation of ROS will occur, leading to an increase in oxidative damage, altering mitochondrial function, neurogenesis, and the normal cell cycle (Spalding et al., 2013; Grimm and Eckert, 2017). These processes leading to neuronal decline during senescence will also contribute to the pathophysiology of neurodegenerative processes (Wang et al., 2019). Among the main markers associated with ageing are elevated oxidative stress, mentioned above, and inflammation (Lee et al., 1999; Finkel and Holbrook, 2000).

Altered cells during ageing acquire a senescence-associated secretory phenotype (SASP) characterized by the secretion of proinflammatory cytokines such as IL-6, IL-1β, TNF-α, and INF-γ, acute phase proteins, chemokines, growth factors and extracellular matrix proteases (Fulop et al., 2018). This SASP phenotype contributes to a sterile, low-grade inflammatory environment by attracting and activating immune system cells (Prata et al., 2018).

The term “inflamm-aging” is used to describe this age-related immune phenotype of inflammation, characterized by a chronic, low-grade pro-inflammatory state, which is related to both activation of the innate immune system and continued exposure to antigens and stress (Franceschi et al., 2000, 2018).

Increased cell ageing and exposure to endogenous infectious agents and harmful signals throughout life imbalance the innate and adaptive immune responses, shifting toward greater involvement of innate immune cells, to the detriment of adaptive immunity based on antigen response (Frasca and Blomberg, 2015).

With ageing, there is an alteration of the adaptive immune response affecting T and B lymphocytes, decreasing the ability to respond to antigens (Gibson et al., 2009; Grubeck-Loebenstein et al., 2009). In addition, during senescence there is an increase in circulating innate immune cells (monocytes/macrophages, neutrophils), as well as an increase in proinflammatory cytokines, indicating an increased innate immune response, which is associated with an increased inflammatory response (Roubenoff et al., 1998; Leng et al., 2005; Tan et al., 2007). Increased serum expression of inflammatory mediators such as C-reactive protein (CRP), IL-6, IL-10, or TNF-α, indicate an activation of the peripheral immune system during ageing and are associated with neurophysiological alterations (Varadhan et al., 2014; Tegeler et al., 2016).

This inflammatory status in the CNS is evidenced by the presence of oxidative stress, increased production and release of pro-inflammatory mediators and the development and progression of neurodegenerative pathologies (Barrientos et al., 2015; von Bernhardi et al., 2015).

In the CNS, as in the peripheral nervous system, there is an age-associated increase in inflammation-related genes such as NFκB, TLR-4, IL-1 receptor and GFAP. In addition, there is an increase in the expression of IL-6, TNF-α, and increased oxidative damage coupled with a reduction in antioxidant capacity (Guest et al., 2014; Primiani et al., 2014). Accumulation of free radicals and increased pro-inflammatory cytokines such as IL-1β and IL-6, combined with decreased expression of anti-inflammatory cytokines such as IL-10 and IL-4, contribute to the maintenance of the senescent inflammatory state (Ye and Johnson, 2001; Nolan et al., 2005; Sierra et al., 2007; Kuzumaki et al., 2010). Along with the above changes, chronic inflammation also compromises the integrity of the BBB, increasing permeability and making the CNS more vulnerable as a result of the entry of cytokines such as IL-1β, IL-6, and TNF-α, and immune cells from the systemic circulation, which are normally strictly regulated. Although these cytokines and immune cells are crucial for neurodevelopment and synaptic plasticity, their excess may contribute to the maintenance of inflammation and chronic activation of CNS immune cells (Steinmetz and Turrigiano, 2010; Niraula et al., 2017; Prieto and Cotman, 2017; Smith et al., 2017).

While low levels of inflammation are associated with healthy neuronal functionality and longevity, high levels of inflammation during ageing are linked to altered CNS homeostasis, physiological imbalance between proinflammatory and anti-inflammatory responses of microglia and astrocytes, as well as increased glial activation (Arai et al., 2015; Walker et al., 2017; Bachiller et al., 2018; Gamage et al., 2020).

#### 2.4.1 Glial changes in ageing

Activation of microglial cells is one of the main features associated with CNS senescence, with changes in morphology, functionality, and molecular expression levels.

During senescence and in the absence of pathology, microglia undergo fragmentation of the cytoplasm, loss of branching, and shortening of cell processes (Streit et al., 2004), leading to a decrease in the microglial arbor area, showing a dendritic, less symmetrical, more elongated, and less circular appearance of the cells (Tremblay et al., 2012; Davies et al., 2017; Soreq et al., 2017; Zöller et al., 2018). In addition, they show reduced motility of their cytoplasmic processes, affecting the immunosurveillance processes (Damani et al., 2011; Lee et al., 2021).

Microglia, during this process, show a less uniform, uneven distribution, with less mobility, showing no alterations in cell number and density (Davies et al., 2017).

Another characteristic of microglia in ageing is the acquisition of a dystrophic phenotype characterized by a total or partial fragmentation of the cytoplasm as well as the cytoplasmic processes. The most common form of dystrophic microglia is those with an unbranched morphology, with an absence of branched cytoplasmic processes and sometimes with the spheroid formation. Abnormally tortuous and short processes may also be present (Streit et al., 2004). In addition, there is also an increase in rod microglia, a phenotype associated with altered axoplasmic outflow during ageing and neurodegeneration (Bachstetter et al., 2017; Davies et al., 2017).

With ageing, altered inflammatory signaling and phagocytic dysfunction lead to changes in the maintenance of homeostasis by microglia, affecting their capacity for immunosurveillance and cellular debris removal (Koellhoffer et al., 2017; Yuan et al., 2020). In addition, they acquire a state of greater sensitization showing an exaggerated inflammatory response, increasing the expression of pro-inflammatory cytokines and inflammation-related factors (Norden and Godbout, 2013; Niraula et al., 2017).

The secretory activity of microglia plays an essential role in the maintenance of synaptic function, the proliferation and maturation of oligodendrocytes and the recruitment of other immune cells. SASP-induced disruption of the microglial secretome results in the alteration of the homeostatic balance necessary for proper neuronal function, leading to an increase in molecules such as IL-6, IL-1β, TNF-α, an increase in inflammasome-related proteins and ROS, as well as increased expression of Nox2 by microglia (Ritzel et al., 2015; Garner et al., 2018; Mejias et al., 2018; Geng et al., 2020). In addition, microglia also undergo a reduction of inflammation suppression-related genes in senescence, such as SIRT1 (gene encoding the protein sirtuin 1), leading to an increase in the expression of pro-inflammatory cytokines such as IL-1β, also contributing to neuronal dysfunction (Cho et al., 2015).

With ageing, activated microglia also show increased expression of antigen presentation-related genes such as MHC-II and CD68, along with suppression of genes such as IL-10 or CD200. They also show increased expression of TLR receptors (1, 2, 4, and 7) and co-receptor CD14 involved in antigen recognition in innate immunity (Frank et al., 2006; Letiembre et al., 2007). In senescence, there is a clear increase in classical markers of microglial activation such as CD206, CD36, CD11b, CD14 as well as MHC-II and PRR. Both the expression of CX3CL1 and its receptor CX3CR1, essential for microglial migration, and CD200 levels that maintain microglia in their quiescent state, are decreased in senescent microglia (Frank et al., 2006; Wynne et al., 2010; Soreq et al., 2017; Zöller et al., 2018). Microglia during senescence also show significant changes in the expression of inflammatory-related genes such as NFκB, C3 or complement factor B (CFB), as well as SASP factors such as IL-1α, IL-1β, IL-6, IL-8, IL-12, TNF-α, C3, CFB, CXCL1, TGF-β, and NO, showing the involvement of senescent microglia in complement activation and immune dysregulation in the retina (Ma et al., 2013; Guillonneau et al., 2017; Ramirez et al., 2017; Sreekumar et al., 2020).

An additional feature of microglial activation in ageing is an increased reactivity to immune system stimuli (Barrientos et al., 2011). Microglia, in response to LPS stimulation, show hyperactivation with high expression of both pro-inflammatory cytokines; IL-1β, and anti-inflammatory; IL-10, extending the reduction in fractalkine receptor expression and decreasing IL-4 receptor expression (Henry et al., 2009; Xue et al., 2021). IL-10 released by microglia binds to IL-10 receptors on astrocytes, which regulate the signaling of the inflammatory phenotype of microglia through the action of TGF-β. Decreased IL-10 receptors on astrocytes as a consequence of senescence lead to dysregulation of microglial activation, showing an exacerbated response (Norden et al., 2014, 2016).

Senescent microglia show dysregulation of phagocytic capacity and may phagocytose live neurons or participate in excessive elimination of synapses via C1q, disrupting synaptic transmission (Hickman et al., 2013; Zhao et al., 2015; Liu et al., 2016). It also shows insufficient capacity to remove apoptotic bodies, protein aggregates and myelin, contributing to the accumulation of potentially toxic agents (Safaiyan et al., 2016). In addition, microglial plasticity to adopt an anti-inflammatory or pro-inflammatory phenotype is also altered as a consequence of age (Harry, 2013).

Several factors contribute to the acquisition by retinal microglia of a pro-inflammatory phenotype during ageing, including altered metabolism and reduced immunomodulatory signals from retinal neurons, such as the CX3CL1-CX3CR1 and CD200-CD200R signaling pathways, resulting in increased microglial activation (Chen et al., 2019).

As with microglial cells, astrocyte activation is also one of the hallmarks of physiological ageing (Nichols et al., 1993; Rodríguez et al., 2014; Robillard et al., 2016). The main features associated with senescent astrocytes include permanent cell cycle arrest, morphological alterations, increased GFAP and vimentin expression, chromatin alterations and the formation of senescence-associated heterochromatic foci (SAHF), increased expression of high-mobility group B (HMGB) proteins, reduced expression of neurotrophic growth factors and increased SASP factors, along with lysosomal and mitochondrial dysfunction (Bitto et al., 2010; Boccardi et al., 2015; Boisvert et al., 2018; Turnquist et al., 2019).

Senescence-associated heterochromatic foci formation and nuclear DNA alteration in senescent astrocytes results in attenuated expression of genes related to cell proliferation, expression of DNA damage response (DDR) markers, as well as increased nuclear size (Souza et al., 2015; Seoane et al., 2017; Hernandez-Segura et al., 2018; Bang et al., 2019; Gorgoulis et al., 2019). Increased HMGB protein expression induces DDR production and cellular inflammation (Enokido et al., 2008).

During senescence, there is a strong parallel between the characteristics of reactive astrocytes and SASP of senescent cells, experiencing both cellular hypertrophy and expression of pro-inflammatory molecules. Thus, during ageing, astrocytes acquire a reactive phenotype associated with low-grade neuroinflammation (Bhat et al., 2012; Clarke et al., 2018). SASP-related gene expression in astrocytes during ageing is considered a consequence of DDR production and is mediated by MAPK and NFκB pathways, as well as HMGB expression (Enokido et al., 2008; Chien et al., 2011; Salminen et al., 2011; Mombach et al., 2015; Seoane et al., 2017). SASP mediators released by senescent astrocytes such as IFN-γ, CXCL10, IL-6, and TGF-β have the ability to induce inflammation and promote microglial activation, contributing to the maintenance of the proinflammatory status characteristic of senescence (Blasko et al., 2004; Bhat et al., 2012; Taylor et al., 2018; Han et al., 2020).

With ageing, there is also an alteration in the signaling of glutamate, the main neurotransmitter in the CNS. Both the levels of glutamate transporters GLAST and GLT-1 released by astrocytes, as well as the expression of GS are altered as a consequence of the ageing process, contributing to increased glutamate excitotoxicity (Bellaver et al., 2017; Boisvert et al., 2018).

Similar to some neurodegenerative diseases, astrocyte activation during senescence is mainly observed in specific areas where there is greater synaptic loss and age-related cognitive decline, such as the hippocampus and frontal cortex frontal (Rodríguez et al., 2016; Matias et al., 2019).

During the normal ageing process, the expression of IL-1α, TNF, and C1q, pro-inflammatory cytokines released by senescent microglia, leads to the activation of astrocytes (Sierra et al., 2007; Hickman et al., 2013; Stephan et al., 2013). Most of the genes expressed by these astrocytes are characteristic of the neurotoxic A1 phenotype, classically induced by neuroinflammatory processes (Zamanian et al., 2012; Liddelow et al., 2017; Clarke et al., 2018). Neurotoxic A1 astrocytes appear with ageing and are associated with age-related cognitive decline (Clarke et al., 2018).

Glial fibrillary acidic protein and vimentin levels, two astrocyte-specific genes directly linked to reactive astrogliosis, also increase during ageing (Nichols et al., 1993; Porchet et al., 2003; Iram et al., 2016; Lye et al., 2019). Increased GFAP and vimentin have been linked to altered cell cycle and reduced cell proliferation and neurogenesis processes in senescence (Larsson et al., 2004). In addition, other signs of astrocyte activation during senescence include cellular hypertrophy with increased cytoplasmic organelles and glial filaments, reduction in the number of astrocytes, expression of proinflammatory cytokines such as IL-1, IL-6, TNF-α, and IL-1β and ROS generation (Nichols et al., 1993; Ramírez et al., 2001; Porchet et al., 2003; Mansour et al., 2008; Rodríguez et al., 2014; Weber et al., 2015; Soreq et al., 2017).

Reactive astrocytes during ageing also show increased expression of genes related to complement system activation such as C3 and C4b, as well as expression of genes related to antigen presentation such as MHC-II and markers of inflammation such as NFκB (Lee et al., 2000; Nolan et al., 2005; Campuzano et al., 2009; Carrero et al., 2012; Iram et al., 2016; Boisvert et al., 2018; Clarke et al., 2018).

#### 2.4.2 Ageing and retina

Retina is also exposed to changes related to cellular ageing such as oxidative stress, cellular hypoxia, DNA alterations, epigenetic alterations, mitochondrial dysfunction, nutrient supply disruption, proteostasis dysfunction and chronic inflammation. The fact that it is constituted mostly of highly specialized post-mitotic cells such as pigment epithelial cells, photoreceptors and RGCs, cells that have a high metabolic activity and oxygen consumption, increases its susceptibility to the effects of senescence (McHugh and Gil, 2018).

Continuous exposure of the retina to visible light contributes to increased oxidative stress. With ageing, the functionality of the pigment epithelium is altered due to the accumulation of waste products such as lipofuscin, which is a potent generator of ROS, contributing to increased oxidative damage. ROS accumulation disrupts proper mitochondrial functionality, leading to reduced ATP synthesis and cell apoptosis. ROS-induced mitochondrial dysfunction alters the mitochondrial respiratory chain and leads to a secondary increase in ROS (Wassell et al., 1999; Davies et al., 2001).

Associated with ageing is also a loss of axons from RGCs, quantified in post-mortem human retinal sections at approximately 3000–7000 axons per year (Harwerth et al., 2008; Calkins, 2013). Although there is variability between different studies, axon loss corresponds to 0.3–0.6% of the total per year between the second and eighth decade of life (Budenz et al., 2007; Harwerth et al., 2008). In the mouse retina, there is a decrease in axon packing density and an increase in axonal diameter accompanied by degenerative changes associated with mitochondrial dysfunction (Zhu et al., 2018). A similar loss of RGCs also occurs in the macula during ageing. Histological samples of human retinas show a decrease in the number of RGCs by 25–43% in the interval between the second and eighth decade of life (Curcio and Drucker, 1993; Blanks et al., 1996).

Retinal glial cells, essential for the maintenance of RGC homeostasis, also undergo age-associated alterations. In senescence, microglia and macroglia acquire a state of chronic activation that results in an autoimmune response, neuroinflammation and altered BRB, ultimately leading to neurodegeneration (Chan-Ling et al., 2007; Xu et al., 2009; Chen et al., 2010).

#### 2.4.3 Ageing and glaucoma

Age is one of the main risk factors associated with the development and progression of glaucoma (Weinreb and Tee Khaw, 2004; Quigley, 2011). The mechanisms involved in physiological cellular senescence make RGCs and their axons more vulnerable to glaucoma-associated changes leading to more rapid cell loss. In aging, pathological mechanisms triggered by increased IOP will affect neuronal tissue which is less resistant to glaucomatous damage (Burgoyne, 2011).

With aging, the lamina cribrosa becomes stiffer as a consequence of collagen deposition and alterations in elastin synthesis associated with stiffening and thickening of the astrocyte basement membranes and extracellular matrix, increasing the susceptibility of the optic nerve head to glaucomatous damage (Albon et al., 2000; Kotecha et al., 2006). These morphological changes could result in reduced blood flow and nutrient supply to RGC axons as they pass through the lamina cribosa (Burgoyne and Downs, 2008; Burgoyne, 2011). In addition, it has been postulated that laminar damage is associated with oxygen deprivation and disruption of the retrograde axonal flow of neurotrophic factors leading to secondary death of RGCs (Quigley et al., 2000; Martin et al., 2006).

Mitochondrial dysfunction is one of the main features of cellular senescence and the cytotoxic effect of ROS accumulation may induce further degeneration of RGCs in glaucoma patients. Increased IOP induces the alteration of the functionality of antioxidant enzymes such as superoxide dismutase, catalase, or glutathione peroxidase, exacerbating the effects of mitochondrial damage (Moreno et al., 2004; Tezel, 2006; Zanon-Moreno et al., 2008).

Chronic activation of glial cells as part of innate immunity occurs as a result of stress factors associated with both increased IOP and aging (Xu et al., 2009). Release of TNF-α, IL-6, IL-18, IL-1β, induces apoptosis of RGCs and degeneration of their axons (Sappington and Calkins, 2008; Munemasa et al., 2010; Tezel et al., 2012). Specifically, levels of TNF-α and its receptor appear higher in the optic nerve head of aged glaucoma patients, contributing to increased axonal neurodegeneration due to increased IOP in conjunction with increased age-related susceptibility (Tezel and Wax, 2000; Yuan and Neufeld, 2000).

**FIGURE 4:**
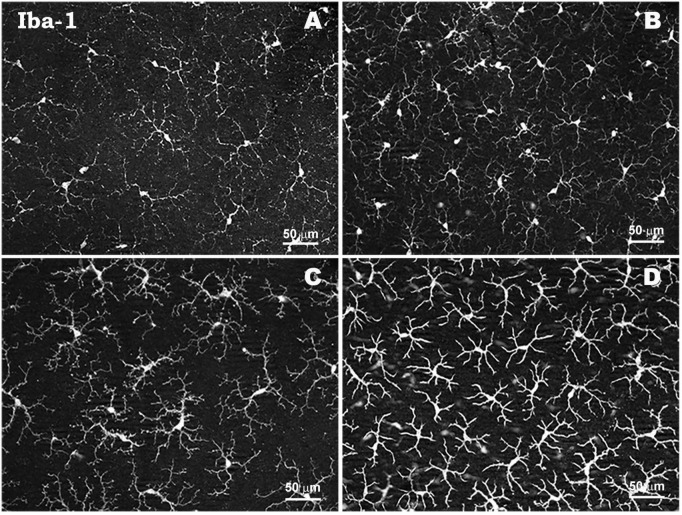
Morphological characteristics of microglial cells in young naïve retina **(A)**, in young retina in response to elevated IOP in a laser-induced ocular hypertension experimental model in mouse **(B)**, in aged naïve retina **(C)**, and in aged retina in response to elevated IOP in a laser-induced ocular hypertension experimental model in mouse **(D)**. Microglia in aged retinas show a more robust aspect with a higher thickening of their soma and retraction of their processes both in normal conditions and in response to IOP increase compared to young retinas. Retinal whole-mount. Iba-1 immunostaining. Modified from Ramírez et al. (2020b).

With aging, the immunosurveillance capacity of microglia is altered, presenting a lower number of extensions and less mobility of these, together with a lower rate of movement to the site of the lesion and a greater inflammatory response (Damani et al., 2011). Microglial activation in aging, characterized by less intense morphological changes in response to elevated IOP, is associated with this senescent altered ability of these cells to respond to damage and a reduced neuroprotective capacity (Orre et al., 2014; Ramírez et al., 2020b; Figure 4).

Evidence suggests that aged animals are more susceptible to RGCs loss in models of acute IOP elevation-induced ischemia, optic nerve crush, or laser-induced ocular hypertension (Ai et al., 2007; Tan et al., 2015; Ramírez et al., 2020b). In addition, in both animal models and humans, aging leads to altered mechanisms regulating the physiological turnover of degraded proteins, triggering increased intracellular accumulation that ultimately leads to apoptosis of RGCs (Wax et al., 2008; Campello et al., 2013).

## 3 Conclusion

In glaucoma, increased IOP and vascular dysregulation are considered two of its main risk factors. Mechanical damage caused by elevated IOP leads to altered axoplasmic flow in RGCs, while reduced blood flow and perfusion pressure at the posterior pole also contribute to neuronal damage. The main treatments for glaucoma are focused on reducing IOP; however, despite controlling IOP, in many cases glaucomatous pathology progresses, demonstrating the existence of other mechanisms involved in neurodegeneration, so that elevated IOP is not the only factor that triggers damage. Mechanisms leading to the neurodegenerative process in glaucoma include ischemia/hypoxia, mitochondrial dysfunction, oxidative stress, and neuroinflammation, among others. Increased oxidative stress along with mitochondrial dysfunction and parainflammatory state that occur during aging constitute another main risk factor related to glaucoma progression.

As in other neurodegenerative pathologies, there is a clear involvement of the immune system in neuroinflammatory processes that occur in glaucoma. The regulation of the immune response in the retina is conducted by glial cells: microglia, astrocytes, and Müller cells. Reactive glial cells, in response to damage caused by OHT, show morphological changes and release cytokines and chemokines that initially contribute to trigger the inflammatory process. Retinal glial population react in response to IOP with the primary objective to isolate and resolve neural tissue damage by providing growth factors and metabolites to damaged RGCs. Thus, a controlled response of glial cells helps to restore the functionality of retinal tissue by exerting a neuroprotective effect; however, a chronic activation of glial cells in response to elevated IOP would contribute to exacerbate the neuroinflammatory response, affecting glial cell function, neuronal support and extracellular glutamate buffering capacity, exerting a neurotoxic effect, being involved in the immune-mediated neurodegenerative process of RGCs and apoptotic death. Glaucoma is a pathology associated with aging. During this process, microglia and astrocytes undergo a state of chronic activation that can lead to neuroinflammation, blood-retinal barrier (BRB) alteration, and neurodegeneration. Consequently, with aging, there is an increased susceptibility to the inflammatory process, resulting in greater neurodegeneration associated with elevated intraocular pressure (IOP) that occurs in glaucoma.

Therefore, immune system modulation and retinal glial cell activation control is an important target in the context of neuroprotection in glaucoma.

## Author contributions

JF-A: Writing – original draft. AR: Writing – original draft. RH: Writing – original draft. JM: Writing – review and editing. ES-G: Writing – review and editing. LE-H: Writing – review and editing. IL-C: Writing – review and editing. LS-P: Writing – review and editing. JS: Writing – original draft. JR: Writing – original draft.
